# Comparative Analysis of Artificial Intelligence Platforms in Generating Post-Operative Instructions for Rhinologic Surgery

**DOI:** 10.1007/s12070-024-05161-1

**Published:** 2024-11-02

**Authors:** Ariana L. Shaari, Shreya Bhalla, Annie Xu, Aman Patel, Andrey Filimonov, Wayne Hsueh, Jean Anderson Eloy

**Affiliations:** 1 Department of Otolaryngology– Head and Neck Surgery, Rutgers New Jersey Medical School, Newark, New Jersey USA; 2 Rutgers Robert Wood Johnson Medical School, New Brunswick, New Jersey USA; 3 Center for Skull Base and Pituitary Surgery, Neurological Institute of New Jersey, Rutgers New Jersey Medical School, Newark, NJ USA; 4 Department of Neurological Surgery, Rutgers New Jersey Medical School, Newark, NJ USA; 5 Department of Ophthalmology and Visual Science, Rutgers New Jersey Medical School, Newark, NJ USA; 6 Department of Otolaryngology and Facial Plastic Surgery, Saint Barnabas Medical Center - RWJBarnabas Health, Livingston, NJ USA

**Keywords:** Rhinology, Patient education, Artificial intelligence

## Abstract

Recently, artificial intelligence (AI) platforms such as ChatGPT and Google Gemini have progressed at a rapid pace. To allow for optimal medical outcomes and patient safety, it is crucial that patients have clearly written post-operative instructions. Patients are increasingly turning to AI platforms for medical information. Our objective was to evaluate the ability of ChatGPT versus Gemini to develop accurate and understandable post-operative instructions for patients undergoing endoscopic sinus surgery (ESS). Postoperative instructions on ESS, balloon sinuplasty, Caldwell Luc surgery, and septoplasty were generated using ChatGPT and Gemini. Measures of readability were calculated. The Patient Education Materials Assessment Tool was utilized to determine Understandability and Actionability. ChatGPT and Gemini were capable of generating understandable post-operative instructions but were poor for actionability and readability. While AI has a promising ability to generate accessible medical information, patients should be aware of its drawbacks especially when seeking postoperative instructions.

## Introduction

Since its release to the public in 2022, artificial intelligence (AI) platform ChatGPT (OpenAI; San Francisco, CA) has gained attention for its ability to aid physicians in patient management and treatment, facilitate clinical research, and help patients understand their health concerns [1]. In 2023, Google Gemini (formerly known as Bard) (Google; Mountain View, CA) was released to the public. AI platforms can serve as valuable resources in postsurgical care by providing patients with information about their recovery [2]. Proper education regarding postoperative care is critical to the success of surgeries, optimizing recovery, and minimizing complications [3]. As AI platforms become increasingly important in modern life, they could serve as a powerful resource to enhance healthcare. However, AI also has a potential drawback to being the primary source where patients obtain their health information.

Correct postoperative care including nasal saline irrigation and steroids is essential in patients undergoing rhinologic surgery [4]. Previous studies have shown that patients have varied understandings of the immediate postoperative period and recovery period [5, 6]. Having clear and understandable postoperative instructions is crucial to ensuring patient safety and optimal surgical outcomes. Patient questions will ideally be answered by their otolaryngologist, but AI platforms could provide guidance and help answer common questions. While AI platforms cannot fully replace clinicians, they are free resources that can provide concise answers and potentially bridge the information gap [2]. 

Patient misconceptions regarding postoperative care and lower health literacy have been associated with worse outcomes; therefore it is crucial to investigate ways to improve patient education [2]. ChatGPT and Gemini have the potential to be excellent resources, but their ability to provide postoperative instructions for rhinologic procedures is poorly investigated. We conducted a comparative analysis between ChatGPT and Gemini to evaluate their accuracy in providing postoperative instructions for endoscopic sinus surgery (ESS), balloon sinuplasty, Caldwell Luc surgery, and septoplasty.

## Methods

Postoperative instructions on the 4 aforementioned rhinologic procedures were generated using ChatGPT 4.0 and Gemini (January 2024). The following prompt was independently entered into each AI platform: “Please write postoperative instructions for a patient who just underwent (rhinology procedure)” for each procedure. The readability of each response was assessed using 2 measures of readability, the Flesch Kincaid Grade Level (FKGL) and Flesch Kincaid Reading Ease (FKRE). The Patient Education Materials Assessment Tool (PEMAT) was utilized to determine Understandability and Actionability. Instructions are deemed understandable when patients of diverse health literacy can process them, and actionable when patients of diverse health literacy can identify steps based on the information. Accepted standards for adequate patient comprehension are FKGL < 6, FKRE > 60, and PEMAT > 80%.

## Results

Eight post-operative instructions were generated. The different AI platforms were not uniformly optimal for patient comprehension (Table 1). ChatGPT had higher understandability than Gemini (mean 98 vs. 92, *p* = 0.038). Gemini had higher actionability than ChatGPT (mean 55 vs. 40, *p* = 0.024) (Table 2). There were no significant differences in FKGL and FKRE between ChatGPT and Gemini (mean 9 vs. 8, mean 56 vs. 52, *p* = 0.093, respectively).

**Table 1:** Evaluations of Readability, Understandability, and Actionability Scores Across 4 Rhinology Procedures from ChatGPT 4.0 and Gemini

Procedure	Artificial Intelligence Platform	Flesch Kinkaid Reading Ease**	Flesch Kinkaid Grade Level***	PEMAT- Understandability****	PEMAT- Actionability****
Endoscopic Sinus Surgery	ChatGPT*	54.9	8.1	90.9	40
	Gemini	54.3	9.1	91.7	40
Balloon Sinuplasty	ChatGPT*	46	9.5	100	40
	Gemini	57.2	8.7	91.7	60
Caldwell Luc Surgery	ChatGPT*	55.2	7.7	100	40
	Gemini	56.5	8.9	91.7	60
Septoplasty	ChatGPT*	51.7	8.5	100	40
	Gemini	57.9	8.6	91.7	60

*ChatGPT 4.0 was utilized; **Scores of 6 are considered to be appropriate for the reading level of an average adult American patient; ***Scores above 60 are considered to have appropriate reading ease for the average adult American patient; ****Scores above 80 are considered appropriate readability and understandability of the average adult patient

**Table 2:** Comparative Evaluation of ChatGPT 4.0 versus Gemini

	ChatGPT*	Gemini	*P*-Value
Flesch Kinkaid Reading Ease**	51.95	56.48	0.094
Flesch Kinkaid Grade Level***	8.45	9.03	0.23
PEMAT-Understandability****	97.73	91.7	0.038*
PEMAT-Readability****	40	55	0.024*

*ChatGPT 4.0 was utilized; **Scores of 6 are considered to be appropriate for the reading level of an average adult American patient; ***Scores above 60 are considered to have appropriate reading ease for the average adult American patient; ****Scores above 80 are considered appropriate readability and understandability of the average adult patient

## Discussion

We found that ChatGPT and Gemini provided adequate postoperative instructions for ESS, balloon sinuplasty, Caldwell Luc surgery, and septoplasty. These instructions may be helpful for patients with varying reading levels and degrees of health literacy. Across all 4 procedures, ChatGPT scored higher for understandability but lower for readability compared to Gemini. There was no significant difference between ChatGPT and Gemini across the 4 procedures for reading ease or grade-level assessment.

In recent years AI models have been introduced into various fields including otolaryngology, notably as a diagnostic tool to optimize patient care and overall has been positively received by providers [7]. Both platforms were capable of generating understandable instructions, but lacked in actionability and readability, meaning that patients with lower degrees of health literacy and English language proficiency would have difficulty understanding and following the instructions [8]. This is particularly significant in the context of rhinology as patients can have a varied understanding and expectations of the postoperative period and expectations of the recovery period [5, 6]. 

While direct interface with medical professionals is ultimately the most important source of patient education, written patient education materials can be valuable. The American Medical Association recommends that written materials be at or below a sixth grade reading level [8]. Lower health literacy has been shown to be correlated with adverse health outcomes compared to those with higher health literacy demonstrating the importance of well written patient instructions [8]. 

Several limitations in our study warrant acknowledgement. Only 4 procedures and 2 AI platforms were analyzed. Drawbacks inherent to both platforms included lack of citations; inability of users to confirm the accuracy of the information; and a knowledge base with a September 2021 end point, excluding the latest data or practice [2]. Although the instructions generated in this study did not provide inaccurate information, previous studies have demonstrated that AI platforms such as ChatGPT can generate false and potentially dangerous information depending on the question asked [1]. Because the same search term were utilized for both search engines, it is possible that different search terms could generate inaccurate information.

## Conclusion

ChatGPT and Gemini generated understandable post-operative instructions but were poor for actionability and readability. While AI hales a promising ability to generate accessible and tailored medical information, patients should be aware of its drawbacks especially when seeking important instructions following rhinologic surgery. Future AI models may be trained on larger and more recent datasets, which may stand to improve safety, reliability, and accuracy of provided clinical information.
